# Characteristics associated with risk taking behaviours predict young people's participation in organised activities

**DOI:** 10.1016/j.adolescence.2016.10.008

**Published:** 2016-12

**Authors:** Britt E. Hallingberg, Stephanie H.M. Van Goozen, Simon C. Moore

**Affiliations:** a Centre for the Development and Evaluation of Complex Interventions for Public Health Improvement, School of Social Sciences, Cardiff University, 1-3 Museum Place, Cardiff, CF10 3BD, United Kingdom; b The Violence and Society Research Group, School of Dentistry, Cardiff University, Heath Park, Cardiff, CF14 4XY, United Kingdom; c School of Psychology, Cardiff University, Tower Building, 70 Park Place, Cardiff, CF10 3AT, United Kingdom

**Keywords:** Organised activities, Extra-curricular activities, Sport, Sensation seeking, Inhibitory control, ALSPAC

## Abstract

Participation in organised activities (OAs) such as sports and special groups can shape adolescent risk taking behaviours. Sensation seeking and inhibitory control play an important role in the emergence of adolescent risk taking behaviours and may explain variations in OA participation as well as inform the development of more effective interventions that use OAs. Data from the Avon Longitudinal Study of Parents and Children (England) were analysed using logistic regression to test whether inhibitory control and sensation seeking predicted participation in OAs at a mean age of 11.7 years (n = 2557) and 15.4 years (n = 2147). At 11 years of age higher sensation seeking predicted participation in any activity, sports and special groups while low inhibitory control predicted less participation in sports. At 15 years of age higher sensation seeking predicted participation in sports and activity breadth. Opportunities to develop targeted interventions aimed at increasing participation are discussed.

## 1 Background

Organised activities (OAs), such as sports, extracurricular activities and other types of youth clubs, have been identified as opportunities to improve young people's health and development (Modecki, Barber, & Eccles, 2014). OA participation is associated with better psychological adjustment (Fredricks & Eccles, 2006a) and improved emotional health (Barber, Eccles, & Stone, 2001); however, not all forms of OA participation protect against risk-taking. Sporting activities in particular are associated with risk taking behaviours such as increased alcohol use, delinquency and violence (Denault et al., 2009, Gardner et al., 2009, Sønderlund et al., 2013).

There are likely fundamental differences between OA participants and non-participants, which contributes to difficulties in disentangling the effects of OA participation (Bohnert, Fredricks, & Randall, 2010). Groups such as young offenders (Hallingberg, Moore, Morgan, Bowen, & Goozen, 2015), children from low-income families (Dearing et al., 2009), and young people with externalising behaviours participate in OAs less often, and are also more likely to use alcohol, exhibit delinquency and violence (Hallingberg et al., 2015), suggesting that those who are least likely to engage in OAs are more likely to engage in these risk behaviours.

Individual characteristics associated with risk taking behaviours, such as sensation seeking and inhibitory control (Casey et al., 2011, Steinberg, 2010), may explain variations in OA participation among young people. Not all risk taking is undesirable (Strang, Chein, & Steinberg, 2013) and reward-seeking tendencies can drive behaviours that are either socially valued (i.e. OA participation) or undesirable (i.e. substance use and delinquency). OAs might mimic the rewards and experiences of undesirable risk taking, although the evidence for this remains debated (Crabbe, 2000, Smith and Waddington, 2004).

Greater sensation seeking is associated with participation in extreme sports, combat activities (Cazenave et al., 2007, D'Silva et al., 2001, Zuckerman, 1994) and more diverse activity participation (D'Silva et al., 2001), while inhibitory control difficulties are comorbid with motor control and developmental problems (Beyer, 1999, Pan et al., 2009) and may reduce opportunities to engage with physical activities (Engel-Yeger and Ziv-On, 2011, Shimoni et al., 2010) and organised play (Cairney et al., 2005).

Although previous studies have examined individual characteristics that are associated with OA participation within ecological frameworks (Dearing et al., 2009, Eisman et al., 2015), sensation seeking and inhibitory control have not yet been investigated. To address this, the current study used a longitudinal British cohort to investigate whether sensation seeking and inhibitory control predicted participation in OAs at 11 and 15 years of age (referred to as early and mid-adolescence respectively). Analyses controlled for respondent's demographic circumstances, previous OA participation, intelligence (IQ) and level of conduct problems.

## 2 Methods

### 2.1 Sample

The Avon Longitudinal Study of Parents and Children (ALSPAC) is a longitudinal population-based cohort in England (see Boyd et al., 2013, Golding, 2004, Golding et al., 2001 for methods). Pregnant mothers in the Bristol-based health districts who were due to give birth between 1st April 1991 to 31st December 1992 were recruited to the study. 14,541 pregnancies were recruited antenatally resulting in 14,676 foetuses. 14,062 of the foetuses results in live births of which 13,988 children were alive at one year of age (Boyd et al., 2013). Participants in this study were limited to those with complete information at a mean age of 11.7 years (SD = 0.1; 48.3% male; 97.7% white; n = 2557) and 15.4 years (SD = 0.2; 47.9% male; 97.3% white; n = 2147). Ethical approval for the study was obtained from the ALSPAC Ethics and Law Committee and local research ethics committees. The study website contains further information on ALSPAC including a searchable data dictionary (http://www.bris.ac.uk/alspac/).

### 2.2 Measures

#### 2.2.1 OA participation

At 8 and 11 years mothers reported whether their child attended a) “any special activity classes (e.g. sports)” and b) any “special groups (e.g. scouts or youth clubs)”; these two categories are referred to as “sports” and “special groups”, respectively, throughout this paper. At 15 years participants reported whether they attended youth clubs, groups, or sports centres on evenings or weekends and indicated the types of activities they participated in: sports, dance activity (keep-fit/aerobics/dance class), music club, drama club, youth club or other. Breadth was measured as the sum of these different activity groups.

#### 2.2.2 Inhibitory control

Inhibitory control was measured at 10 years using the stop-signal paradigm (Logan, Cowan, & Davis, 1984). The procedure outlined by Handley, Capon, Beveridge, Dennis, and Evans (2004) was used to administer and score the task. Four blocks of trials were presented: 30 primary task trials, 24 practice trials and two experimental blocks consisting of 48 trials each. The number of correct trials inhibited when the stop signal occurred 150 ms before participant's mean reaction time was used and the top ten percent of participants who failed the most number of trials were coded as low inhibitory control.

#### 2.2.3 Sensation seeking

At 11 and 13 years sensation seeking was assessed by the intensity subscale of Arnett's Inventory of Sensation Seeking (AISS, Arnett, 1994; Cronbach's alpha age 11 = 0.568; age 13 = 0.611), a ten item Likert-type scale. Higher scores indicated greater sensation seeking. To make the questionnaire more age-appropriate the original questionnaire item: “In general, I work better when I'm under pressure” was replaced with: “I think it's fun and exciting to perform or speak before a group”. The AISS has been validated as a measure of risk taking behaviour in adolescent populations (Arnett, 1994, Roth and Herzberg, 2004) and in contrast to other measures, the AISS is “conceived as being influenced by a biological predisposition which interacts with the social environment” and does not contain items associated with physical strength, antisocial or norm-breaking behaviour (Roth & Herzberg, 2004, p. 206).

#### 2.2.4 Conduct problems

At 11 years of age the conduct disorder subscale of the Strength and Difficulties Questionnaire (Goodman, 1997) was used to indicate sub-optimum behavioural outcomes for conduct problems. This subscale consisted of five Likert-type scale items (Cronbach's alpha = 0.559). The questionnaire and subscale has well-established reliability in terms of internal consistency and retest stability (Goodman, 2001). Similar to previous studies using the ALSPAC cohort (Hibbeln et al., 2007) the prorated score was used to create a dichotomous measure. The low tails of the distribution of gender-specifics scores (closest to 10%) were categorised as having conduct problems. At 15 years, 13 Likert-type scale items from the Edinburgh Study of Youth Transitions in Crime (Smith & McVie, 2003), were used to measure conduct problems. Similar to previous procedures using this cohort (MacArthur et al., 2012), participants who reported any engagement in antisocial behaviours in the past year were categorised as having conduct problems (Cronbach's alpha = 0.779).

#### 2.2.5 IQ

IQ was estimated using the shortened Wechsler Intelligence Scale for Children, 3rd UK edition (Wechsler, Golombok, & Rust, 1992) at 8 years using a total score of the verbal and performance subscales (Cronbach's alpha = 0.728 and 0.517 respectively) scaled according to participant's age. At 15 years the Wechsler Abbreviated Scale of Intelligence (Wechsler, 1999) measured IQ using a total score from the vocabulary and matrix reasoning subscales (Cronbach's alpha = 0.823 and 0.531 respectively) scaled according to participant's age.

#### 2.2.6 Analytical approach

Logistic regressions compared non-participants to participants in: a) any OA, b) any sport and c) any special groups at 11 years of age. At 15 years of age non-participants were compared to participants in a) any OA and b) a sport. Ordered logistic regression investigated predictors of breadth. Clusters of sibling pairs within the sample were controlled for in analyses and all analyses were carried out on STATA IC 11 software.

## 3 Results

Analyses predicted the likelihood of participation in any OA, a sport and a special group at 11 years (see Table 1). Higher levels of sensation seeking predicted participation in any OA (OR = 1.04, 95% CI = 1.02, 1.07, p = 0.002), sports (OR = 1.05, 95% CI = 1.02, 1.08, p = 0.001) and special groups (OR = 1.04, 95% CI = 1.01, 1.07, p = 0.003) while low inhibitory control predict less participation in sports (OR = 0.63, 95% CI = 0.43, 0.92, p = 0.018).

Analyses then predicted the likelihood of participation in any OA, a sport and breadth of OA participation at 15 years (see Table 1). Higher levels of sensation seeking predicted participation in sports (OR = 1.03, 95% CI = 1.01, 1.05, p = 0.011) and breadth (OR = 1.02, 95% CI = 1.0004, 1.04, p = 0.045). Inhibitory control did not predict OA participation.

Due to a low Cronbach's alpha for the IQ measure at age 15, sensitivity analyses were conducted using models without an IQ measure and models with the IQ measure at age 8. Higher levels of sensation seeking predicted participation in any OA, sports and breadth in models without IQ (OR = 1.02, 95% CI = 1.0001–1.04, p = 0.049; OR = 1.03, 95% CI = 1.01, 1.06, p = 0.003; OR = 1.02, 95% CI = 1.005, 1.04, p = 0.012, respectively) and in models with IQ at age 8 (OR = 1.02, 95% CI = 1.004–1.05, p = 0.018; OR = 1.04, 95% CI = 1.02, 1.06, p = 0.001; OR = 1.03, 95% CI = 1.008–1.05, p = 0.005, respectively). Inhibitory control did not predict participation in any OA, sports and breadth in models without IQ (OR = 0.90, 95% CI = 0.67–1.22, p = 0.492; OR = 0.86, 95% CI = 0.61–1.22, p = 0.393; OR = 0.94, 95% CI = 0.70–1.26, p = 0.679, respectively) and in models with IQ at age 8 (OR = 0.96, 95% CI = 0.70–1.30, p = 0.779; OR = 0.93, 95% CI = 0.65–1.32, p = 0.677; OR = 0.997, 95% CI = 0.74–1.34, p = 0.986, respectively).

Likelihood ratio tests determined whether sensation seeking and inhibitory control improved model fit at 11 and 15 years. Adding these variables significantly improved the model when predicting any participation (χ^2^ (2) = 13.4, p = 0.001), participation in sports (χ^2^ (2) = 17.0, p < 0.001) and participation in special groups (χ^2^ (2) = 9.8, p = 0.007) at 11 years. These variables significantly improved the model when predicting sport participation (χ^2^ (2) = 6.7, p = 0.267), but not when predicting any participation (χ^2^ (2) = 2.6, p = 0.268) or breadth of participation (χ^2^ (2) = 4.3, p = 0.118) at 15 years.

## 4 Discussion

Sensation seeking and inhibitory control, individual characteristics important for risk taking (Casey et al., 2011) are also important for OA participation. Increases in sensation seeking may facilitate young people's autonomy by enhancing their “motivation to seek out incentives and new experiences” (Somerville, Jones, & Casey, 2010) and “channelled into a wide range of activities and pursuits” such as hobbies and interests (Dahl, 2004, p. 18). As OAs offer unique learning opportunities distinct from school work and unstructured leisure time (Larson, 2000), they may be a platform to engage young people in socially valued activities that provide rewarding experiences and independence.

Sports are frequently used as diversionary activities and are viewed as socially-acceptable forms of risk taking, yet young people with inhibitory control difficulties were less likely to participate in sports during early adolescence. Inhibitory control deficits may contribute to difficulties in following rules, to heightened emotional reactivity and therefore increased levels of aggression in OAs (Johnson & Rosen, 2000). Young people with challenging behaviours such as conduct disorder, which is associated with response inhibition (Oosterlaan, Logan, & Sergeant, 1998) and comorbid with ADHD (Biederman, Newcorn, & Sprich, 1991) may be sensitive to figures of authority, such as sport coaches, if viewed as too authoritarian (Haudenhuyse, Theeboom, & Coalter, 2012). They may choose not to participate due to a lack of school-based identity or differences in peer group affiliations, such as less academically-oriented or prosocial peers who are more likely to participate in OAs (Eccles & Barber, 1999). They may also be more likely to be excluded if their participation conflicts with creating a safe and welcoming environment for others (Kelly, 2011) and educational institutions may ban participation among young people with challenging behaviours as a form of punishment (Power, Taylor, Rees, & Jones, 2009). Future research should seek to understand if and why there are unique barriers to sport that present to these young people and how they might be related to these individual characteristics. Investment in social programmes may alleviate economic and similar barriers to OA participation but may be less effective against barriers due to these challenging behaviours. Addressing barriers using an ecological framework may therefore be more effective than simply targeting one factor at one level (McLeroy et al., 1988, Vella et al., 2014).

OAs have has been shown to protect against harmful alcohol use among young people with early pubertal timing (Modecki et al., 2014) and may protect against risk taking behaviours for high sensation seekers. The current results also highlight sensation seeking as a self-selection factor (Fredricks and Eccles, 2006b, Larson, 2000). Future research should test the mediating role of sensation seeking to understand its impact on the relationship between OA participation patterns and associated risk taking behaviours such as alcohol use (Peretti-Watel, 2009).

This study sample was less representative of those with lower household income, lower social class as well as ethnic minorities. Inhibitory control was only measured once during early adolescence, and since it develops linearly with age (Casey et al., 2011, Steinberg, 2010), it may have been a weak indicator of later OA participation. Additionally, the conduct disorder scale from the Strength and Difficulties questionnaire as well as the AISS and WASI had low internal reliability for this sample size, increasing the amount of error. There was some uncertainty surrounding the relationship of sensation seeking and any OA participation during adolescence in the absence of the WASI measure in the model and this is an area that needs further attention. Nevertheless, the findings highlight variation in OA participation based on individual characteristics associated with risk taking and can inform efforts that seek to increase OA participation among vulnerable groups of young people.

## Figures and Tables

**Table 1:** Odd ratios and 95% confidence intervals for the measures used to predict participation patterns in early and mid-adolescence.

Measures	Participation patterns in early adolescence								
	Any activity^a^			Sport^b^			Special group^c^		
	OR	95% CI	*P*	OR	95% CI	*P*	OR	95% CI	*P*
Sensation seeking	1.04	[1.02, 1.07]	0.002	1.05	[1.02, 1.08]	0.001	1.04	[1.01, 1.07]	0.003
Low inhibitory control	0.69	[0.48, 1.01]	0.053	0.63	[0.43, 0.923]	0.018	0.79	[0.52, 1.18]	0.248

Measures	Participation patterns in mid-adolescence								
	Any activity^d^			Sport^e^			Breadth^f^		
	OR	95% CI	*P*	OR	95% CI	*P*	OR	95% CI	*P*
Sensation seeking	1.02	[0.996, 1.04]	0.115	1.03	[1.01, 1.05]	0.011	1.02	[1.0004, 1.04]	0.045
Low inhibitory control	0.94	[0.69, 1.28]	0.692	0.93	[0.65, 1.33]	0.693	1.00	[0.75, 1.35]	0.999

*Note.* All analyses adjusted for age, gender, ethnicity, mother's social class, household weekly income, adults in household, estimated IQ and conduct problems.

a n = 2557.

b n = 2256.

c n = 1599.

d n = 2147.

e n = 1802.

f n = 2145.
